# The pro-tumorigenic cytokine IL-32 has a high turnover in multiple myeloma cells due to proteolysis regulated by oxygen-sensing cysteine dioxygenase and deubiquitinating enzymes

**DOI:** 10.3389/fonc.2023.1197542

**Published:** 2023-05-29

**Authors:** Martin Kastnes, Kristin Roseth Aass, Siri Anshushaug Bouma, Charlotte Årseth, Muhammad Zahoor, Mariia Yurchenko, Therese Standal

**Affiliations:** ^1^Centre of Molecular Inflammation Research, Department of Clinical and Molecular Medicine, Norwegian University of Science and Technology, Trondheim, Norway; ^2^Department of Molecular Medicine, Institute of Basic Medical Sciences, University of Oslo, Oslo, Norway; ^3^Department of Hematology, St.Olavs University Hospital, Trondheim, Norway

**Keywords:** multiple myeloma, proteasome, ubiquitin (UB), IL-32, T cells, deubiquitinase (DUB), oxygen stress, ADO

## Abstract

IL-32 is a pro-inflammatory cytokine expressed by several types of cancer cells and immune cells. Currently, no treatment targeting IL-32 is available, and its intracellular and exosomal localization make IL-32 less accessible to drugs. We previously showed that hypoxia promotes IL-32 expression through HIF1α in multiple myeloma cells. Here, we demonstrate that high-speed translation and ubiquitin-dependent proteasomal degradation lead to a rapid IL-32 protein turnover. We find that IL-32 protein half-life is regulated by the oxygen-sensing cysteine-dioxygenase ADO and that deubiquitinases actively remove ubiquitin from IL-32 and promote protein stability. Deubiquitinase inhibitors promoted the degradation of IL-32 and may represent a strategy for reducing IL-32 levels in multiple myeloma. The fast turnover and enzymatic deubiquitination of IL-32 are conserved in primary human T cells; thus, deubiquitinase inhibitors may also affect T-cell responses in various diseases.

## Introduction

Multiple myeloma (MM) is a malignancy of terminally differentiated plasma cells. MM cells are home to the bone marrow, where the microenvironment supports cancer cell proliferation and survival (1). The bone marrow is hypoxic, and MM cells overexpress HIF1α, which may turn the oxygen stress into a benefit by increasing the cancer cells’ ability to disseminate (2, 3).

MM is an incurable disease, but newer therapy regimens have substantially improved survival (4). The frontline treatment today includes proteasome inhibitors. Proteasomal degradation is central to the removal of excess and dysfunctional proteins in all cells. In addition, proteasomal degradation is important in regulating processes that must be timed and happen quickly, such as cell cycle progression and stress responses. Most cytokines are regulated by transcription and mRNA stabilization, and the intracellular cytokine level is kept low due to rapid secretion rather than by degradation by the proteasome (5, 6). The leaderless interleukin IL-1β is an exception to this rule, as it can be regulated by proteasomal degradation (7–9).

Interleukin (IL)-32 is involved in the progression of several cancer types and a range of inflammatory diseases (10, 11). IL-32 mRNA expression is a prognostic factor in MM and is upregulated in cancer cells of patients that have relapsed from treatment (12, 13). *In vitro*, IL-32 is induced upon hypoxia in MM cell lines, and in patient samples, IL-32 mRNA expression correlates with a hypoxic signature (12). Depletion of IL-32 from three different MM cell lines reduces proliferation and cell survival as well as tumor growth *in vivo* (13). Due to its pro-inflammatory properties, IL-32 is classified as a cytokine although it has no sequence homology with the other cytokine family members (14). It lacks the signal peptide that directs a protein to the conventional secretion pathway but can still promote inflammation by extracellular signaling (10). This may be due to the release of IL-32 upon cell death and the secretion of IL-32 in extracellular vesicles (12). Importantly, and in contrast to most other cytokines, IL-32 plays an important intracellular role (10). For example, we previously demonstrated that IL-32 interacts with the mitochondria to promote oxidative phosphorylation (13).

IL-32 mRNA and protein expression are regulated by two different oxygen sensing systems: HIF1α (12) and cysteamine (2-aminoethanethiol) dioxygenase (ADO) (15), respectively, which suggests that IL-32 is involved in cellular oxygen stress responses. Understanding how IL-32 protein stability and turnover are regulated is important to unravel this unconventional cytokine’s role in health and disease and to develop therapeutics that can target IL-32 intracellularly. We here show that IL-32 protein is synthesized at a high rate and has a short half-life. We also demonstrate that IL-32 protein stability is regulated by ADO and deubiquitinases (DUBs). Our results support the hypothesis that ubiquitination and proteasomal degradation are the primary pathway of degradation of the IL-32 protein. Thus, DUB inhibitors can be used to reduce IL-32 protein levels and may be a therapeutic option in diseases where IL-32 plays an important role.

## Materials and methods

### Cells and culture conditions

The MM cell line JJN-3 and INA-6 were kind gifts from Dr. Jennifer Ball (University of Birmingham, UK) and Dr. Martin Gramatzki (University of Erlangen-Nuremberg, Erlangen, Germany), respectively. OH-2 and IH-1 were established in our laboratory (16, 17). The cells were cultured in RPMI-1640 medium with fetal calf serum or human serum as previously described (18). For T-cell isolation and activation, human PBMCs were isolated from buffy coat using Lymphoprep (Stem Cell Technologies, Vancouver, Canada) according to the manufacturer’s protocol. PBMCs were incubated overnight in full medium (RPMI medium with 10% heat-inactivated fetal calf serum (FCS) with gentamicin) before T cells were isolated with Dynabeads Untouched Human T Cells Kit (Thermo Fisher Scientific, Waltham, MA, USA). T cells were cultured in full medium supplemented with IL-2 at 30 U/ml (PeproTech/Thermo Fisher Scientific, waltham, MA, USA). To increase the number of T cells and to increase IL-32 expression, T cells were activated with Dynabeads™ Human T-Activator CD3/CD28 (Thermo Fisher) with a 1:1 cell:bead ratio for 72 h before bead removal. The cells were cultured at 37°C in a humidified atmosphere containing 5% CO_2_.

### Treatment with proteasome inhibitors, DUB inhibitors, and cycloheximide

For cycloheximide (CHX) protein degradation chase assays, CHX was used at a final concentration of 5 or 7 µg/ml, while proteasomal and autophagic inhibitors MG132 and bafilomycin A1 (Sigma-Aldrich, St. Louis, MO, USA) was used at a concentration of 20 μM and 90 nM, respectively. Carfilzomib and bortezomib (Selleck Chemicals, Houston, TX, USA), were both used at a final concentration of 50 nM. DUB inhibitors and the concentrations used are found in **Supplementary Table S1**.

### ADO siRNA experiments

Using buffer R (Amaxa Nucleofector Kit R, Lonza, Basel, Switzerland) and program T-001 on Nucleofector device (Amaxa Biosystems, Cologne, Germany), 2 µM ADO ON-TARGETplus siRNA (L-015855-01-0005) and ON-TARGETplus Non-targeting Pool (D-001810-10-05) siRNA from Dharmacon were transfected into JJN-3 cells by electroporation. The cells were seeded into 12-well plates 24 h after siRNA transfection and cultured overnight in normoxic (20% O_2_, 5% CO_2_) or hypoxic conditions (2% O_2_, 5% CO_2_) before 5 µg/ml CHX was added and harvested at a timepoint of 0 min/h. Plates were then placed back in the normoxic incubator (reoxygenation and normoxia) and the hypoxic incubator (hypoxia), and the cells were harvested at the indicated time points.

### Real-time quantitative PCR

Total RNA was isolated using the RNeasy Mini Kit (Qiagen, Hilden, Germany), and complementary DNA (cDNA) was synthesized from total RNA using the High Capacity RNA-to-cDNA kit (Applied Biosystems/Thermo Fisher Scientific, Waltham, MA, USA). qPCR was performed using StepOne Real-Time PCR System and TaqMan Gene Expression Assays (Thermo Fisher Scientific) with standard settings (2′ 50°C, 10′ 95°C, 40 cycles at 95°C for 15 s, 1′ 60°C). The comparative Ct method was used to estimate relative changes in IL-32 gene expression using *β*-actin and GAPDH as housekeeping genes. Probes were as follows: human IL-32 (Hs00992441_m1) and housekeeping gene GAPDH (Hs99999905_m1) housekeeping gene *β*-actin (Hs0160665_g1) both from Thermo Fisher Scientific.

### Immunoblotting

Cells were lysed in lysis buffer (50 mM Tris–HCl, 1% NP40, 150 mM NaCl, 10% glycerol, 1 mM Na_3_VO_4_, 50 mM NaF, and a complete protease inhibitor (Roche Diagnostics, Mannheim, Germany). Lysates were denatured in 1× NuPage LDS sample buffer supplemented with 0.1 mM DTT for 10 min at 70°C before they were separated on 4%–12% Bis‐Tris polyacrylamide gel. Proteins were transferred to a nitrocellulose membrane using the iBlot Dry Blotting System (Invitrogen, Camarillo, CA, USA). Membranes were blocked using 5% bovine serum albumin (Sigma-Aldrich) in Tris‐buffered saline with 0.01% Tween, followed by overnight incubation with the primary antibodies. Detection was performed using horseradish peroxidase (HRP)-conjugated antibodies (DAKO, Glostrup, Denmark) and developed with Super Signal West Femto Maximum Sensitivity Substrate (Thermo Fisher Scientific, Rockford, IL, USA). Images were obtained with LI‐COR Odyssey Fc and analyzed using Image Studio Software (LI‐COR, Lincoln, NE, USA). The following antibodies were used: anti-IL-32 (#AF3040, R&D Systems, Minneapolis, MN, USA), anti-β-actin (#4967, Cell Signaling Technology, Danvers, MA, USA), anti-GAPDH (# ab8245, Abcam, Cambridge, UK), anti-HA (#H6908, Sigma-Aldrich), anti-ubiquitin (# 13-1600, Thermo Fisher), and anti-ADO (ab134102, Abcam).

### Confocal imaging

JJN-3 cells were seeded in poly-l-lysine coated 96-well glass-bottom plates and treated with carfilzomib for 4 h at a concentration of 100 nM at 37°C before being fixed with 4% PFA for 10 min at RT. After quenching for 10 min with 50 mM NH_4_Cl, cells were washed with PBS and then permeabilized using 0.15% triton X-100 in PBS. Cells were washed with 0.02% Tween before blocking with 20% human serum (HS) in PBS. Anti-IL-32 (R&D) antibody were diluted to 2 μg/ml in 1% HS and 0.02% Tween and was left on cells overnight at 4°C. The next day, secondary antibody chicken anti-goat IgG (H + L) antibody Alexa Fluor 647 (2 μg/ml) in 1% HS, 0.02% Tween was added for 30 min before leaving cells in Hoechst (Thermo Fisher Scientific) in PBS (2 μg/ml) for imaging.

### Ubiquitin pulldown assay

JJN3 cells were incubated overnight in hypoxic (2% O_2_, 5% CO_2_) conditions. Before harvest, the cells were stimulated for 4 h with 20 µM MG-132 in normoxic conditions. Approximately 10 million JJN-3 cells were lysed in lysis buffer (1% NP40, 10% glycerol, 150 mM NaCl, 50 mM Tris-HCl at pH 7.5, 1 mM EDTA, 100 mM *n*-ethylmaleimide, 1 mM Na_3_VO_4_, 50 mM NaF, and a complete protease inhibitor (Roche Diagnostics, Mannheim, Germany)). Ubiquitinated proteins were pulled down with tandem ubiquitin binding entities (TUBEs) conjugated to magnetic beads with the kit UM411M: UbiTest Magnetic TUBE Elution Kit (LifeSensors) according to the manufacturer’s protocol.

### HA-Ubiquitin transfection

HA-Ubiquitin (19) was a gift from Edward Yeh (Addgene plasmid # 18712 (12); http://n2t.net/addgene:18712; RRID : Addgene_18712). Five micrograms of HA-ubiquitin plasmid was transfected into JJN-3 cells by electroporation using buffer R (Amaxa Nucleofector Kit R, Lonza) and program T-001 on the Nucleofector device (Amaxa Biosystems, Cologne, Germany). Cells were cultured overnight in hypoxic conditions (2% O_2_, 5% CO_2_) and then harvested for IL-32 immunoprecipitation.

### IL-32 immunoprecipitation

IL-32 antibody (R&D) was conjugated to M-270 beads according to the manufacturer’s instructions, using Dynabeads Antibody Coupling Kit (Thermo Fisher) and 10 µg of resuspended IL-32 antibody/mg of beads. JJN-3 KO and WT cells were incubated in hypoxia for 24 h before lysis with 4× pellet volume RIPA buffer (1% CHAPS, 50 mM Tris, 150 mM NaCl, 1 mM Na_3_VO_4_, 50 mM NaF, and a complete protease inhibitor (Roche Diagnostics)) for 1 h at 4°C on rotation. Lysate was incubated with IL-32 antibody-conjugated beads for 1 h at 4°C. After washing four times with PBS, beads were resuspended in 1× NuPage LDS sample buffer supplemented with 0.1 mM DTT and heated for 10 min at 70°C before beads were removed and the sample proceeded to immunoblotting.

## Results

### IL-32 is degraded through the ubiquitin–proteasome pathway

**Figure 1:**
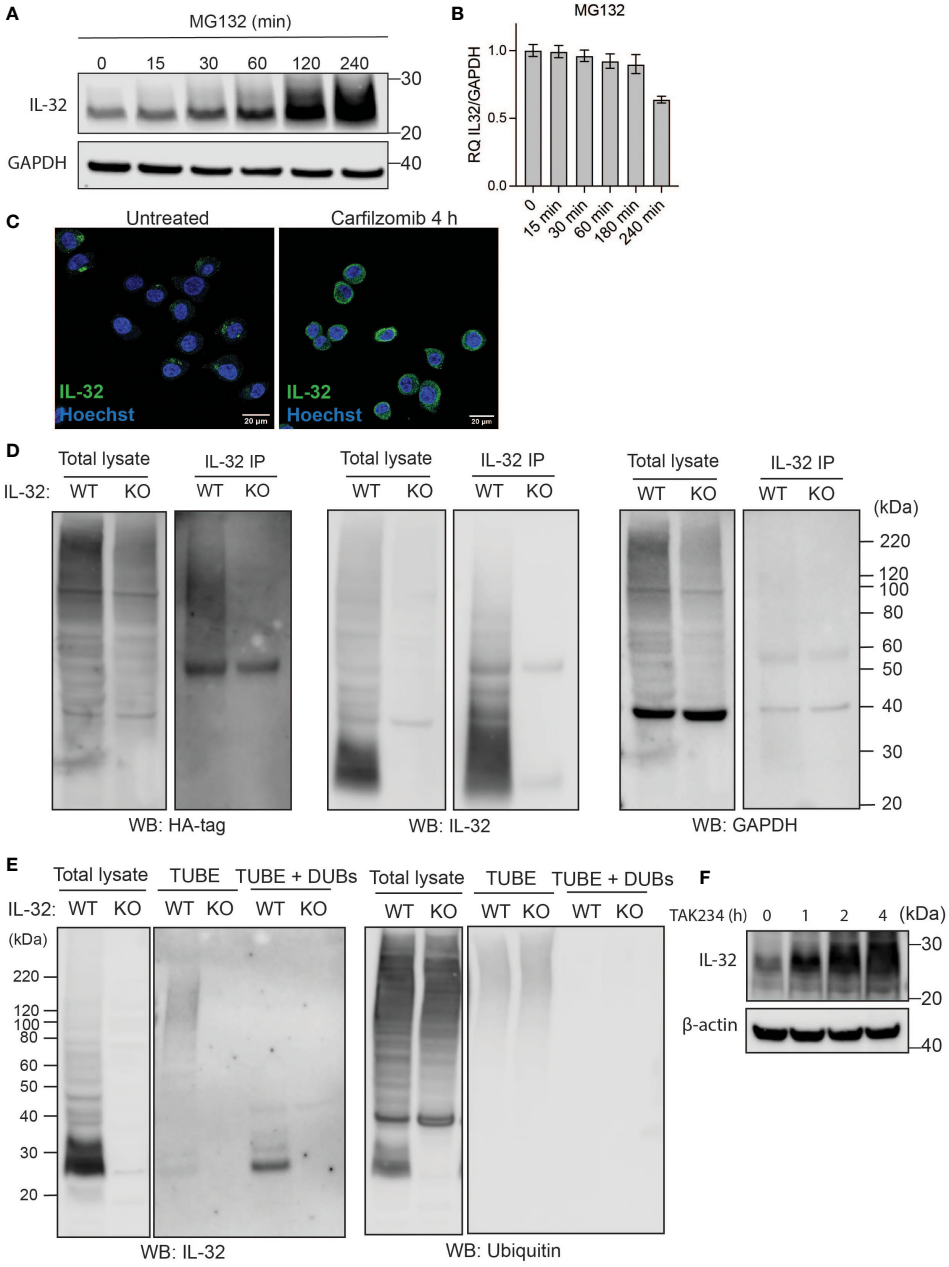
IL-32 is degraded through the ubiquitin-proteasome pathway. **(A, B)** JJN3 cells were treated with 20 µM MG-132 and harvested at the indicated time points. IL-32 protein levels were analyzed by **(A)** Western blotting, and **(B)** IL-32 mRNA was assessed by qPCR using GAPDH as housekeeping gene. The bars show the mean RQ of IL-32 ± SD. **(C)** Confocal images of JJN3 cells treated for 4 h with 100 nM carfilzomib and stained with IL-32 antibody (green) and Hoechst (blue). **(D)** JJN3 cells were transfected with HA-ubiquitin plasmid and incubated in hypoxia overnight before the cells were harvested and IL-32 immunoprecipitation was performed. Protein levels of HA-ubiquitin, IL-32, and GAPDH loading control were evaluated on WB. Total lysate and IP samples from the same membrane are shown with different brightness/contrast. **(E)** JJN3 cells were incubated overnight in hypoxia before they were stimulated with MG-132 for 4 h and harvested for TUBE assay of ubiquitinylated proteins. The presence of IL-32 and ubiquitin protein in TUBE pulldown was assessed by WB. DUB treatment for reversal of polyubiqutinylation was included to validate ubiquitin/TUBE pulldown. Total lysate and TUBE samples from the same membrane are shown with different brightness/contrast. **(F)** JJN3 cells were stimulated with 2 μM TAK234 before the cells were harvested, and IL-32 levels were analyzed by WB. **(A–F)** One representative experiment out of three is shown.

We previously identified interaction partners of IL-32 in MM cells by IL-32 pulldown of cell lysates, followed by mass spectrometry (13). Two of the identified binding partners were the proteasomal alpha ring-subunit PSMA3 and the E3 ubiquitin ligase WWP2, which may suggest that IL-32 is ubiquitinylated and degraded by the proteasome. Indeed, when we treated the MM cell line JJN-3 with the proteasome inhibitor MG132, we observed an accumulation of IL-32 protein that was evident after 2 and 4 h of treatment (**Figure 1A**), while IL-32 mRNA did not increase (**Figure 1B**). IL-32 protein was similarly stabilized by MG132 treatment in three other myeloma cell lines: INA-6, IH-1, and OH-2 (**Supplementary Figures S1A, B**). Treatment with carfilzomib and bortezomib, proteasomal inhibitors that are used in the treatment of MM, had a similar effect: accumulation of IL-32 protein (**Supplementary Figure S1C**) with no change in mRNA expression (**Supplementary Figure 1D**). Treatment with bafilomycin A also led to some accumulation of IL-32 protein (**Supplementary Figures S1C, D**), indicating that IL-32 may also be partially degraded through an autophagosomal pathway. Confocal imaging of carfilzomib-treated JJN-3 cells showed that IL-32 was localized at distinct puncta in the cytosol in untreated cells while abundantly present in the cytosol after 4 h with proteasome inhibitor treatment (**Figure 1C**).

Proteins are targeted for proteasomal degradation by poly-ubiquitination executed by E1, E2, and E3 ligases (20). To see if IL-32 is ubiquitinated, we transfected JJN3 IL-32 CRISPR/Cas9 KO (13) and WT cells with a HA-ubiquitin plasmid and performed a pulldown of IL-32. Staining for the HA tag on the Western blot showed a high molecular weight smear on the WT pulldown samples with no smear on the KO pulldown sample (**Figure 1D**), which supports that IL-32 is poly-ubiquitinated. To demonstrate the endogenous ubiquitination of IL-32, we purified the polyubiquitinylated proteome of JJN-3 IL-32 KO and WT cells using magnetic bead-coupled TUBEs. IL-32 was detected upon TUBE-pulldown in WT cells but not in KO cells, supporting that it is polyubiquitinylated (**Figure 1E**). When treating the TUBE-pulldown sample with DUBs, only a ~27-kDa IL-32 band appeared in the pulldown samples, validating TUBE pulldown of ubiquitinated IL-32. IL-32 also accumulated when the E1 ubiquitin-activating enzyme was inhibited using the small molecule E1 inhibitor TAK234 (21), further supporting that IL-32 is degraded in a ubiquitin-dependent manner (**Figure 1F**).

### IL-32 has a high protein turnover

**Figure 2:**
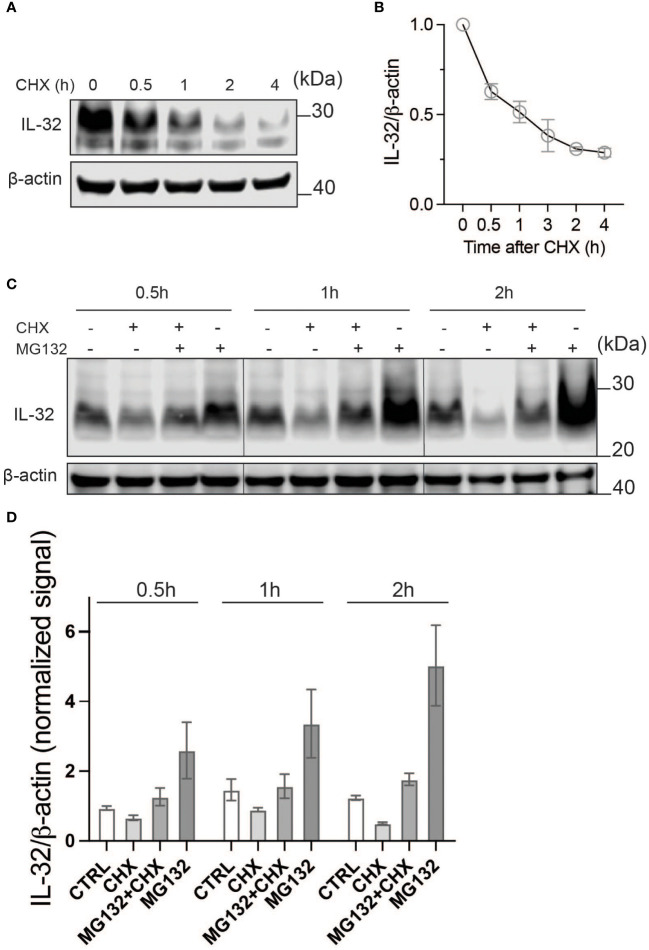
IL-32 has a high protein turnover. **(A)** JJN-3 cells were treated with CHX and harvested at the indicated time points. IL-32 protein levels were analyzed by WB. **(B)** Kinetics of IL-32 degradation in JJN3- cells. IL-32 protein signal intensity was quantified and normalized to loading control in *n* = 6 CHX chase experiments. The mean ±SEM is shown here. **(C)** JJN-3 cells were treated with 5 μg/ml CHX and 20 μM MG132 alone and in combination and harvested at the indicated time points. The figure shows one representative WB from *n* = 3 independent experiments. **(D)** Signal intensities of IL-32 and β-actin were quantified from *n* = 3 independent experiments performed as in **(C)**, and the values from treated samples at each time point were normalized relative to the control sample.

To look into the kinetics of IL-32 protein turnover, we treated JJN-3 cells with CHX to inhibit protein translation. Treating JJN-3 cells with CHX showed a 40% reduction of IL-32 protein after 30 min, with a further decrease at later time points (**Figures 2A, B**). In a similar manner, IL-32 protein was quickly reduced after CHX treatment in INA-6, IH-1, and OH-2 cells (**Supplementary Figure S2**), supporting a high protein turnover in these cells as well. Combining CHX with the proteasome inhibitor MG132 in JJN-3 cells efficiently maintained the steady-state level of IL-32 protein, suggesting that proteasomal degradation is responsible for the short half-life of IL-32 (**Figure 2C**). By comparing IL-32 protein levels in MG132-treated samples (degradation inhibited) with CHX and MG132 (translation and degradation inhibited), we could assess how protein translation contributes to IL-32 protein turnover (**Figures 2C, D**). The level of IL-32 protein in cells treated with MG132 alone for 30 min was roughly doubled compared with cells treated with the combination of CHX and MG132, while at 2 h the difference was nearly threefold (**Figure 2D**), demonstrating that IL-32 mRNA is rapidly and continuously translated. In conclusion, intracellular IL-32 protein in MM cells is maintained at a steady state by a fast and coordinated translation and degradation.

### IL-32 protein half-life is regulated by oxygen-sensor ADO

**Figure 3:**
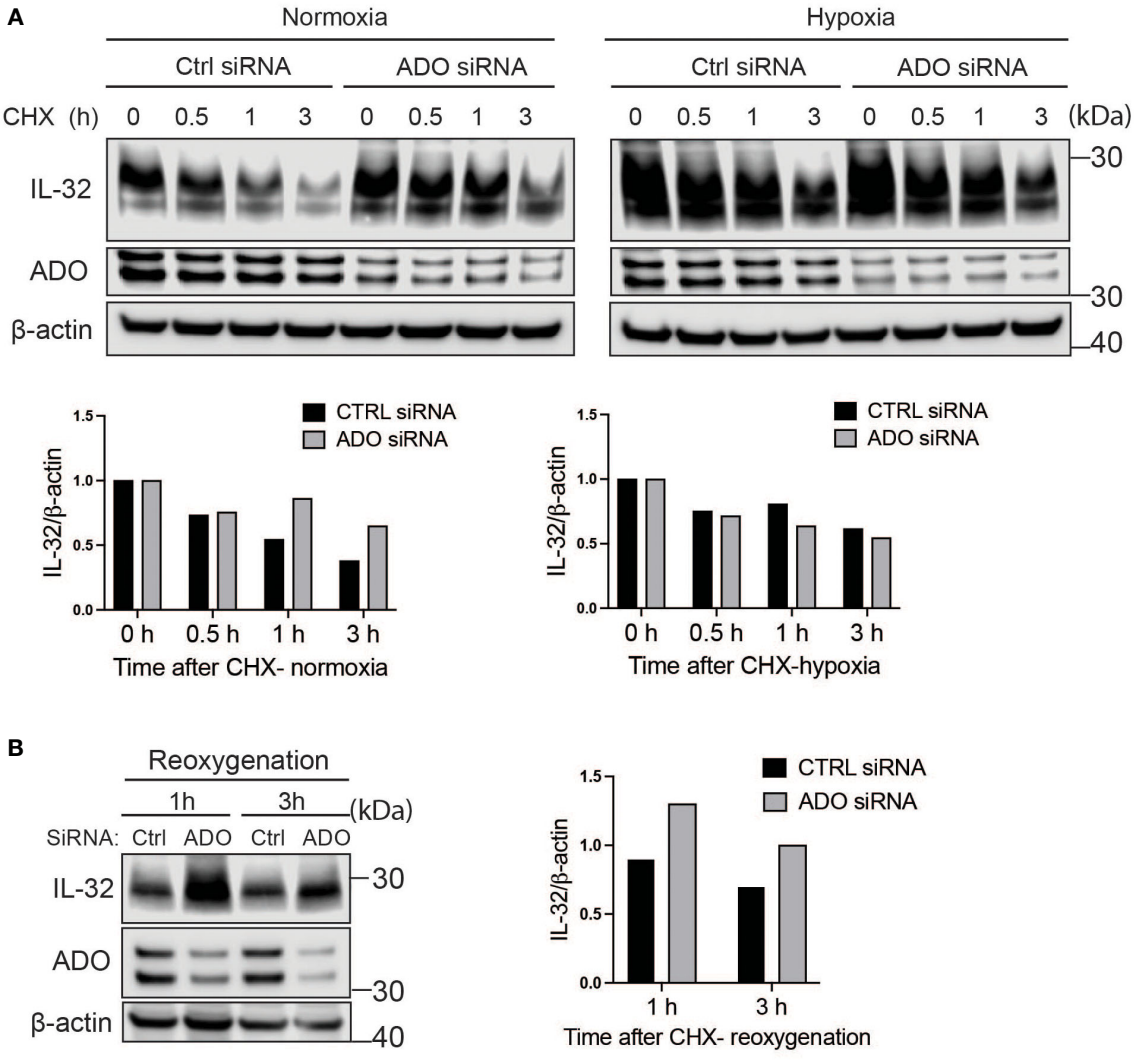
IL-32 protein half-life is regulated by the oxygen sensor ADO. **(A)** JJN-3 cells were transfected with ADO- and nontargeting Ctrl siRNA. After 24 h, the cells were seeded and cultured overnight in normoxia or hypoxia before being treated with 5 μg/ml CHX and the IL-32 CHX chase assay in normoxia and hypoxia. One representative WB of IL-32 and ADO siRNA-treated cells of *n* = 5 independent experiments is shown. **(B)** JJN-3 cells were transfected with ADO and nontargeting Ctrl siRNA. After being transfected for 24 h, the cells were cultured overnight in hypoxia before being treated with 5 µg/ml CHX and reoxygenized in normoxic culture conditions. Cells were harvested at indicated time points.

We previously showed that IL-32 is upregulated in hypoxia and that transcriptional activation is regulated by HIF1α (12). ADO is a recently discovered oxygen-sensing cysteine dioxygenase in humans that targets proteins for proteasomal degradation through the N-degron pathway (15). In the same study, IL-32 protein stability was shown to be regulated by ADO in the RKO cancer colon cell line (15). Ubiquitylation targets proteins for degradation in the proteasome through the N-degron pathway (22). Thus, we investigated if ADO regulates IL-32 protein levels in MM cells in response to changing oxygen levels. We treated cells with CHX to assess the degradation of IL-32 independently of translation. ADO was highly expressed in MM cells, and ADO siRNA only partly depleted the ADO protein (**Figure 3A**). Regardless, in cells with reduced ADO expression, we observed a translation-independent increase in IL-32 protein levels compared to control cells, supporting that IL-32 is regulated by ADO-dependent degradation (**Figure 3A**). ADO catalytic activity is dependent on oxygen availability. As expected, in hypoxia (2% O_2_) in the presence of CHX, there was no difference in IL-32 protein levels between ADO knockdown cells and control cells (**Figure 3A**). Reactivation of ADO by reoxygenation (20% O_2_) of hypoxic ADO knockdown cells and control cells in the presence of CHX demonstrated that IL-32 was more rapidly degraded in control cells (**Figure 3B**), supporting the hypothesis that ADO is involved in the regulation of IL-32 in MM cells during oxygen fluctuations.

### IL-32 is stabilized by deubiquitinases

**Figure 4:**
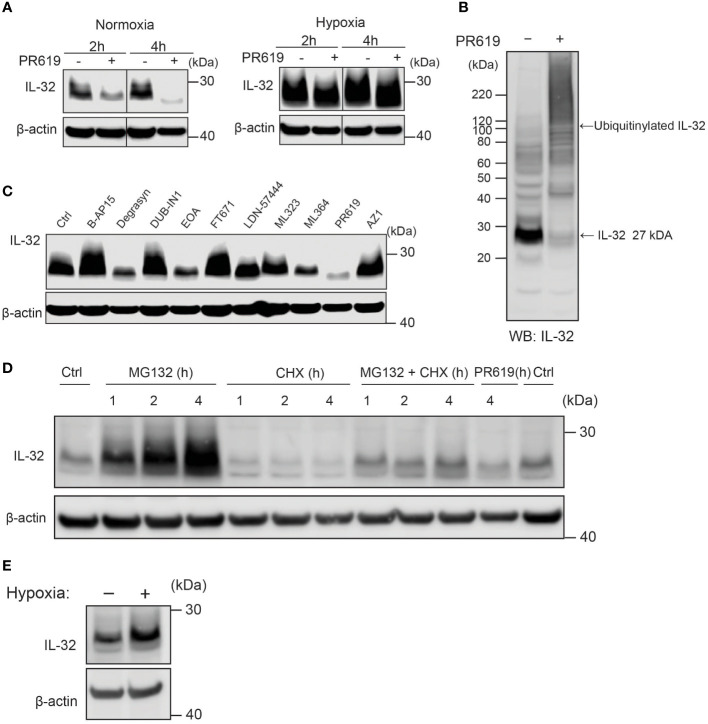
IL-32 is stabilized by deubiquitinases. **(A)** JJN3 cells were stimulated with PR619 (30 μM during normoxia and hypoxia before the sample was harvested at indicated time points. The figure shows representative WB of IL-32 protein levels of *n* = 3 independent experiments. **(B)** JJN3 cells were incubated overnight in hypoxia before they were moved to normoxia for 4 h with and without PR619 stimulation. Cells were harvested, and lysates were processed in the presence of a NEM-DUB inhibitor. Ubiqutinylated and nonubiqutinylated 27 kDA IL-32 proteins were assessed by WB. **(C)** JJN3 cells were stimulated with a panel of DUB inhibitors (see **Supplementary Table S1** for concentrations) for 4 h, and IL-32 protein levels were analyzed by WB. Shown here is the representative WB of *n* = 3 independent experiments. **(D)** Human primary T cells isolated from healthy blood donors were treated with CHX (5 µg/ml), MG132 (20 µM), and PR619 (30 µM) and harvested at the indicated time points. Shown here is the representative WB from experiments with *n* = 3 donors. **(E)** Human primary T cells isolated from healthy blood donors were cultured overnight in hypoxia before IL-32 protein levels were assessed by WB. A representative WB from experiments with three different donors is shown here.

Despite oxygen-dependent degradation of IL-32 by ADO, MM cells express high IL-32 protein levels in normoxia (10, 12). We thus asked how high levels of IL-32 protein are maintained in physiological oxygen levels despite rapid ubiquitylation and proteasomal degradation. Ubiquitylation is a process that can be reversed by DUBs which remove ubiquitin from target proteins (23). Removal of ubiquitin will reduce the degradation of the target protein. Using the broad targeting nonselective DUB inhibitor PR-619, we observed a clear reduction in IL-32 protein after 2 and 4 h (**Figure 4A**), indicating that DUBs counteract ubiquitin-dependent degradation of IL-32 in MM cells in normal oxygen conditions. In hypoxia, the differences between PR-619 and untreated cells were smaller, indicating that there is less deubiquitination of IL-32 in hypoxia (**Figure 4A**). This is in line with reduced ADO-dependent IL-32 ubiquitylation in hypoxia. PR-619 treatment increased the amount of high molecular weight ubiquitinylated IL-32 and decreased nonubiquitinylated IL-32 at 27 kDa in JJN-3 cells (**Figure 4B**), supporting that DUBs remove ubiquitin on IL-32. DUBs are a large and diverse set of about 100 enzymes divided into six families, and PR-619 is a nonspecific, broad-spectrum DUB inhibitor (24). We, therefore, performed a DUB-inhibitor screen to explore which DUBs could remove ubiquitin from IL-32. Using a panel of more specific DUB inhibitors (**Supplementary Table S1**), we observed a large reduction of IL-32 protein levels with degrasyn (targets USP9x, USP5, USP14, UCH37), EOAI3402143 (targets USP9x/USP24 and USP5), and ML364 (targets USP2) (**Figure 4C**). Thus, several different DUBs can potentially contribute to the removal of ubiquitin from IL-32.

### Fast turnover of IL-32 by proteasomal degradation is controlled by deubiquitinases in primary T-lymphocytes

To investigate if protein regulation of IL-32 is conserved in cells of the lymphocyte lineage, we assessed IL-32 protein level in PBMC-derived primary human T cells isolated from healthy donors. Indeed, we observed protein turnover of IL-32 within a similar time frame in activated primary human T cells as in MM cells. IL-32 protein showed accumulation upon MG-132 treatment after 1 h of stimulation and was depleted by CHX treatment within the same time interval (**Figure 4D**). Furthermore, IL-32 was reduced by PR-619 treatment, indicating that DUBs actively remove ubiquitin from IL-32, also in T cells. IL-32 was also slightly increased in hypoxic T cells (**Figure 4E**).

## Discussion

We have previously demonstrated that IL-32 is increased in hypoxia by HIF1-dependent translation (12). We here show that IL-32 has a tightly regulated and short half-life in normoxia by being continuously ubiquitinated and degraded. We further found that IL-32 protein is degraded in the proteasome and that the process is regulated by oxygen-sensing cysteamine dioxygenase ADO with subsequent ubiquitination. DUBs of the ubiquitin-specific peptidase (USP) family remove ubiquitin from IL-32, thereby increasing its stability. Thus, the IL-32 protein is regulated at multiple levels and can be quickly altered in response to cellular stress.

Rapid adaptations to reduced oxygen are crucial for cell survival in hypoxic environments. The proteasome is involved in the tightly controlled degradation of key oxidative stress proteins, including NRF2, HIF1, and NF-κB (25). By inhibiting translation, we observed that IL-32 has a half-life of 1 h and could thus be considered a short-lived protein (26). The rapid intracellular turnover of IL-32 in MM cells, influenced by changes in oxygen levels, suggests a role for this cytokine in cellular/oxidative stress responses.

We previously showed that IL-32 increases the MM cells’ metabolic fitness and promotes proliferation and cell survival (13). Importantly, first-line treatment for MM patients includes proteasome inhibitors (4). Treatment with bortezomib and carfilzomib accumulated IL-32 intracellularly, which may have been followed by an extensive release of IL-32. Extracellular IL-32 has been shown to modulate the MM BM microenvironment in several ways, e.g., by inducing osteoclast differentiation and bone destruction (12), by enhancing the production of pro-tumorigenic IL-6 production by stromal cells (27), and by promoting the formation of immunosuppressive macrophages (28). Thus, due to its direct effects on tumor cells and the induction of a pro-tumorigenic microenvironment, targeting IL-32 could be beneficial in a subgroup of patients. No drug targeting IL-32 is, however, currently available. Antibody-based targeting strategies may be of limited value since IL-32 acts intracellularly (13) and is protected inside extracellular vesicles when secreted (12). Our results support the idea that DUB inhibitors may be useful in reducing IL-32 protein levels.

DUBs play an important role in hematological malignancies (24). MM cells express different DUBs, and these are suggested as potential treatment targets and are currently exploited in clinical trials (VLX1570, USP14 inhibitor, NCT02372240) (29, 30). Results from our screen of a broader panel of DUB inhibitors indicate that several of the USP-DUBs are involved in the deubiquitination of IL-32 in MM cells. Indeed, some of our candidate DUBs have been previously studied in MM. For example, USP9x is linked to poor prognosis in myeloma and the regulation of MCL-1 (31, 32), while USP5 was shown to regulate c-MAF levels (33). A potential drug for USP5 in MM is mebendazole (34).

We found that regulation of IL-32 by ubiquitination-dependent proteasomal degradation and reversing the process by deubiquitinating enzymes are also found in healthy cells of the lymphocyte lineage, as demonstrated with primary T cells. IL-32 is highly expressed by activated T cells (35–38). DUBs are currently exploited as therapeutic targets in different cancers (23, 39–41), but they may also influence the cancer microenvironment, including T cells (42). Our data show that the use of proteasome inhibitors and DUB inhibitors affects IL-32 protein levels in T cells, which should be kept in mind when using DUB inhibitors in cancer treatments.

In conclusion, we show that IL-32 protein has a fast turnover in MM cells and primary T-lymphocytes. The oxygen-sensing enzyme, ADO, regulates the degradation of IL-32 in MM cells, while proteasomal degradation of IL-32 is continuously counteracted by the action of DUBs. DUB inhibitors as treatment in MM may thus also be beneficial to reduce intracellular levels of IL-32.

## Data availability statement

The raw data supporting the conclusions of this article will be made available by the authors, without undue reservation.

## Author contributions

MK and KA designed and conducted experiments, analyzed data and wrote the manuscript. SB, CÅ, MZ and MY conducted experiments and analyzed data, TS designed and supervised the study, analyzed data and wrote the manuscript. All authors contributed to the article and approved the submitted version.

## Supplementary material

Supplementary Figure 1 IL-32 is degraded by the proteasome. **(A)** The myeloma cell lines INA-6, OH-2 and IH-1 were stimulated with MG132 (15 *μ*M) and harvested at the indicated time points. IL-32 protein expression was analyzed by WB **(B)** INA-6, OH-2 and IH-1 were treated with MG132 (15 *μ*M) and harvested at the indicated time points. IL-32 mRNA was assessed by qPCR using *β*-actin as housekeeping gene. **(C)** JJN3 was treated with Cafilzomib (50nM), bortezomib(50nM) and Bafilomycin A1 (90 nM) and harvested at indicated time-points. IL-32 protein levels were analyzed by WB. Figure show representative WB of n=3 independent experiments. **(D)** IL-32 mRNA isolated from the same samples as in **(C)** was assessed by qPCR using GAPDH as housekeeping gene. The figure shows mean RQ of IL-32 ± SD of the representative experiment shown in **(C)**.

Supplementary Figure 2 IL-32 has a high protein turnover. IH-1, OH-2 and INA-6 cells were treated with 7 µg/ml CHX and the cells were harvested at the indicated timepoints. IL-32 protein expression was evaluated by western blot.
