# Supplementary material for: The pro-tumorigenic cytokine IL-32 has a high turnover in multiple myeloma cells due to proteolysis regulated by oxygen-sensing cysteine dioxygenase and deubiquitinating enzymes
Source: Front Oncol. 2023 May 29;13:1197542. doi: 10.3389/fonc.2023.1197542 (PMC10258340; doi:10.3389/fonc.2023.1197542)
Supplement: Supplementary file 1 [file Table_1.docx]

**Supplementary Table 1: DUB inhibitors**
PR-619 acquired from SigmaAldrich (St. Louis, MO, USA), all other DUB inhibitors acquired from MedChemExpress (NJ, USA).

| **Inhibitor** | **Proposed Target DUBs** | **Working concentration** | **Cat. No.** |
| --- | --- | --- | --- |
| [PR-619](https://www.sigmaaldrich.com/NO/en/product/mm/662141) | Unspecific | 30 µM | 662141 |
| [ML364](https://www.medchemexpress.com/ML364.html) | USP2 | 10 µM | HY-100900 |
| [FT671](https://www.medchemexpress.com/FT671.html) | USP7 | 1 µM | HY-107985 |
| [DUB-IN-1](https://www.medchemexpress.com/DUBs-IN-1.html) | USP8 | 2 µM | HY-50736 |
| [Degrasyn](https://www.medchemexpress.com/WP1130.html) | USP9x, USP5, USP14, UCH37 | 5 µM | HY-13264 |
| [EOAI3402143](https://www.medchemexpress.com/EOAI3402143.html) | USP9x/USP24 and USP5. | 3 µM | HY-111408 |
| [USP25/28 inhibitor AZ1](https://www.medchemexpress.com/usp25-28-inhibitor-az1.html) | USP25/28 | 10 µM | HY-117370 |
| [LDN-57444](https://www.medchemexpress.com/LDN-57444.html) | UCHL1, UCHL3 | 10 µM | HY-18637 |
| [ML-323](https://www.medchemexpress.com/ML-323.html) | USP1-UAF1 | 20 µM | HY-17543 |
| [b-AP15](https://www.medchemexpress.com/b-AP15.html) | UCHL5, Usp14 | 1 µM | HY-13989 |
