# Supplementary figures and images for: The pro-tumorigenic cytokine IL-32 has a high turnover in multiple myeloma cells due to proteolysis regulated by oxygen-sensing cysteine dioxygenase and deubiquitinating enzymes
Source: Front Oncol. 2023 May 29;13:1197542. doi: 10.3389/fonc.2023.1197542 (PMC10258340; doi:10.3389/fonc.2023.1197542)

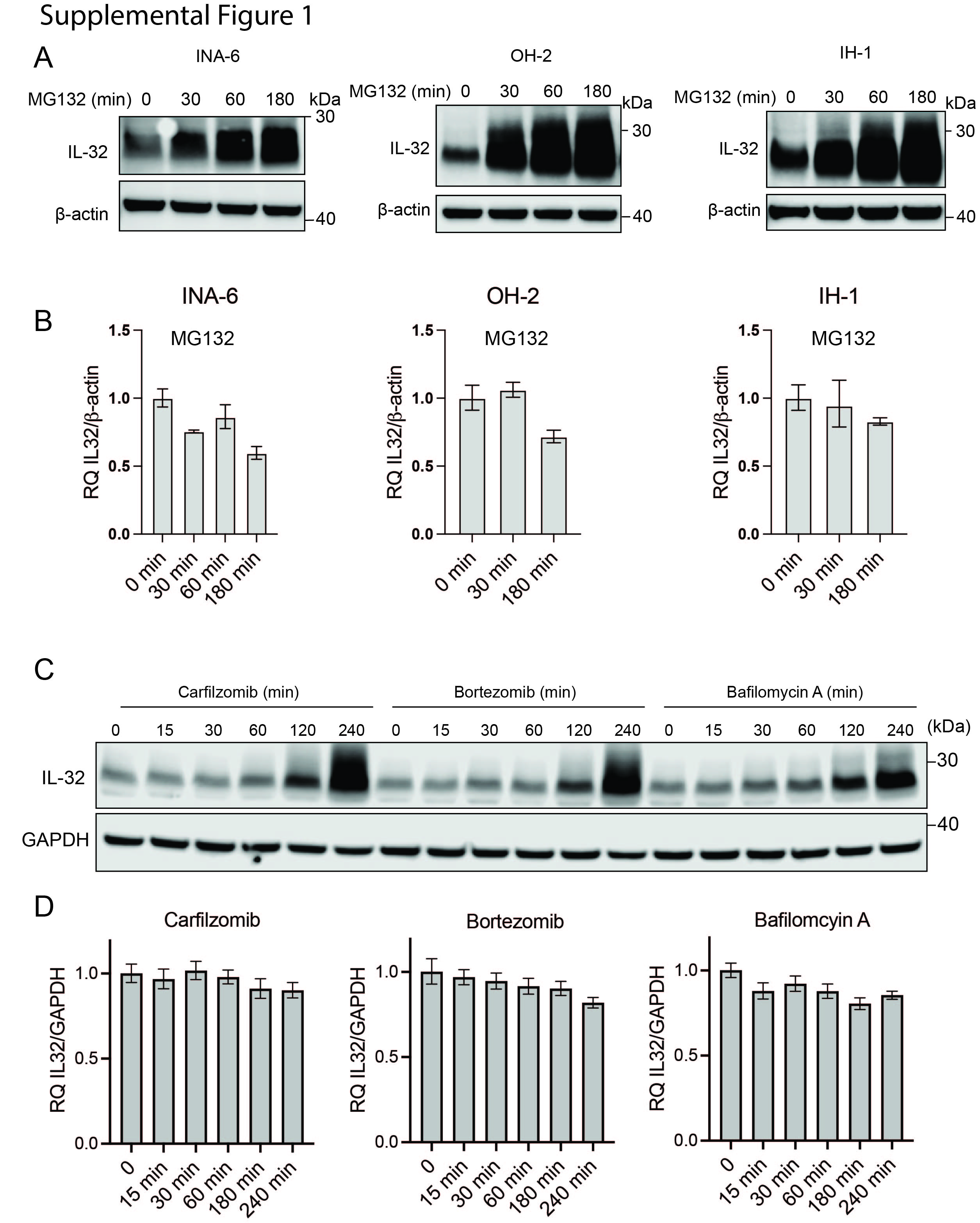

Supplement: Supplementary Figure 1 — IL-32 is degraded by the proteasome. (A) The myeloma cell lines INA-6, OH-2 and IH-1 were stimulated with MG132 (15 μM) and harvested at the indicated time points. IL-32 protein expression was analyzed by WB (B) INA-6, OH-2 and IH-1 were treated with MG132 (15 μM) and harvested at the indicated time points. IL-32 mRNA was assessed by qPCR using β-actin as housekeeping gene. (C) JJN3 was treated with Cafilzomib (50nM), bortezomib(50nM) and Bafilomycin A1 (90 nM) and harvested at indicated time-points. IL-32 protein levels were analyzed by WB. Figure show representative WB of n=3 independent experiments. (D) IL-32 mRNA isolated from the same samples as in (C) was assessed by qPCR using GAPDH as housekeeping gene. The figure shows mean RQ of IL-32 ± SD of the representative experiment shown in (C). [file Image_1.jpeg]

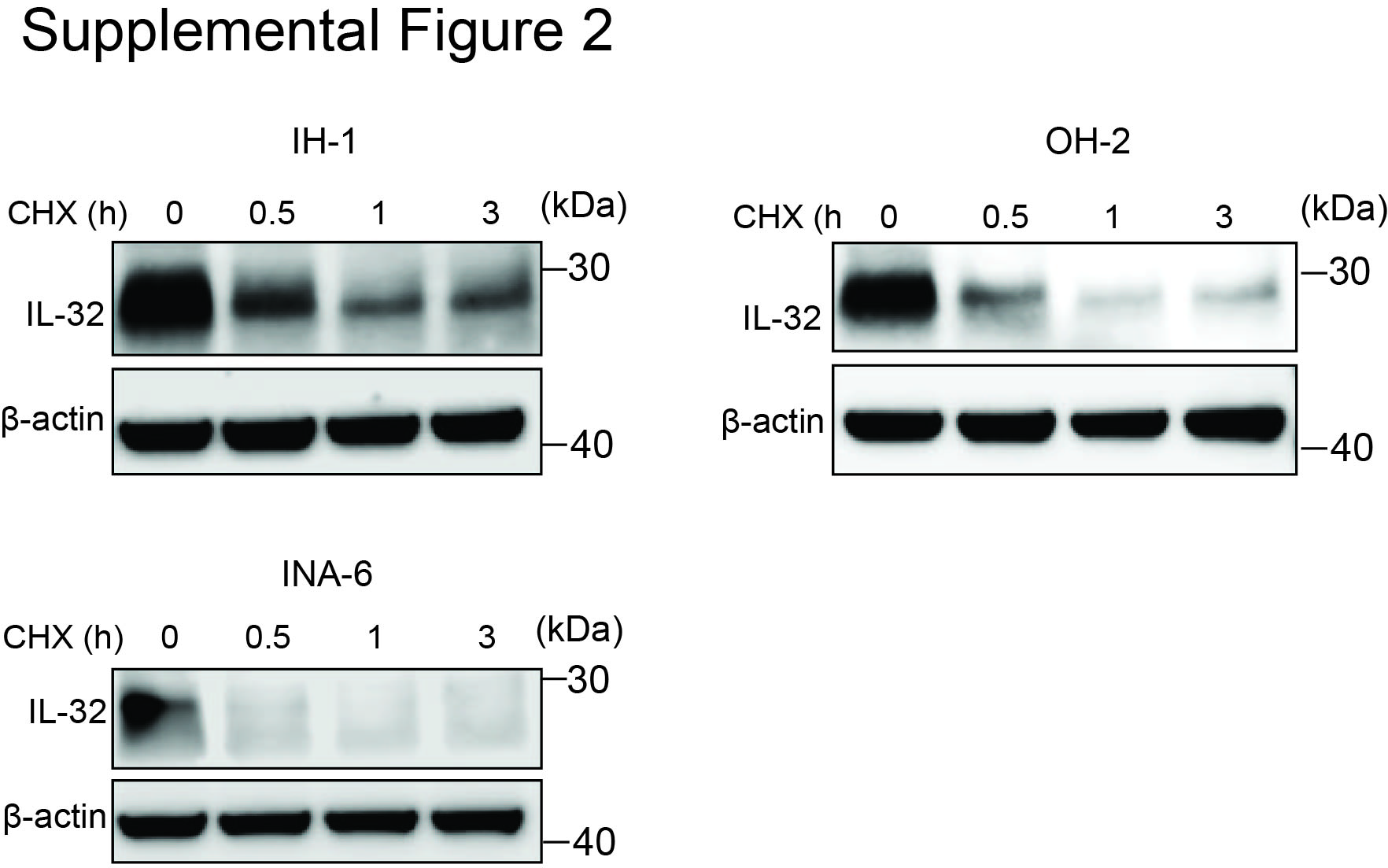

Supplement: Supplementary Figure 2 — IL-32 has a high protein turnover. IH-1, OH-2 and INA-6 cells were treated with 7 µg/ml CHX and the cells were harvested at the indicated timepoints. IL-32 protein expression was evaluated by western blot. [file Image_2.jpeg]
